# Leprosy

**Published:** 1889-11-30

**Authors:** 


### The Practitioner's Outlook and Retrospect.

[*The Editor will be glad to receive offers of co-operation and contributions from members of the profession. There is no doubt that much valuable material full of interest and instruction is at present lost to science. This is largely so in the case of* (1) *the younger and less-known members of the hospital staff,* (2) *those connected with cottage hospitals, and* (3) *the great body of medical practitioners not attached to any institution. The co-operation of all is cordially invited to enable the Editor to make* The Hospital *of the utmost possible use and value to the profession. Specialists willing to review books on their special subjects are invited to communicate this fact at once. All letters should be addressed to* The Editor, The Lodge, Porchester Square, London, W.]

### MEDICINE.

### LEPROSY.

At a recent meeting of the Bombay Medical and Physical Society, the subject of leprosy was discussed at some length, the meeting having been adjourned for this purpose twice. As the members of the Bombay Medical and Physical Society are civil and military practitioners, both European and Indian, constantly seeing leprosy, there is probably no body of men in the world whose opinions should carry greater weight. The paper originating this prolonged discussion was read by Dr. Balchandra Krishna. The author said that leprosy prevails most among males, that it might last for few or many years, and that people affected often live to old age. As regards hereditary transmission, Dr. Krishna stated the disease is hereditary to a very great extent. Statistics were quoted in support of this, and Dr. Krishna remarked: "The malady seems to be transmitted through an old hereditary taint. . . . . This fact seems to be lost sight of when it is said that leprosy is on the decrease in a particular province, owing to segregation." In estimating the causes of leprosy, one must be very careful to distinguish between production and propagation. Leprosy cannot be attributed to any one special cause, but its propagation depends upon heredity, inoculation and intermarriage. After some remarks on pathology and diagnosis, the author drew certain conclusions, among which are that leprosy is endemic; transmission by heredity; the communicability of tuberculated and mixed forms of leprosy in the ulcerated stage by transmission of pus to the abraded surface of another person's skin; the non-communicability by aerial infection; that the segregation of lepers, although some check, would not stamp out the disease. Dr. Krishna next referred to the recent legislation of the Government of India on the subject. He alluded to the lecture on leprosy given some time ago in London by Sir William Moore, and agreed with Sir W. Moore, who stated that segregation may diminish leprosy in India, but would not eradicate it. Syphilitic persons are at times equally loathsome as lepers. Phthisis destroys thousands more than leprosy does. The former has long been known to be contagious; there is some evidence that the latter is also, yet no one proposes syphilitic people and phthisical people should be segregated. Leper asylums should be supplied for poor helpless lepers. But well-to-do people should not be dealt with in the same manner, for they do not show themselves, and are not likely to prove dangerous to the community. Dr. Krishna thinks there has been an unwarrantable scare on the subject of leprosy, and he entreated the meeting to "beware of leprophobia." Dr. Temuljee Narriman said he concurred in the main with the conclusions of Dr. Krishna, but he saw no reason why the subject should be now so much agitated. To his mind, hygienic and climatic conditions were the principal factors to be considered. Taking an analogy from the vegetable world, if we planted a seed it is not the earth alone which imparted growth and life. Light, heat, and other essentials are required. It is the same with leprosy. Food, climatic influences, and particular states of the body have a great deal to do with the development of leprosy. But fish food as a principal factor, is quite out of the question. As against the view of contagion, Dr. Narriman gave three instances of only one leper in a family. He also gave an instance of a woman marrying four leper husbands, and remaining healthy. Still, he thought leprosy was contagious, but only in the sense that syphilis is contagious. He would like the poor lepers to have a home to shelter them, but he did not think segregation ought to be compulsory for those "who could afford to lie in their own beds." Segregation would do an injury to those only little affected. Put away among advanced lepers, they would stand a serious chance of having their disease increased. Dr. R. N. Khory said the public were agitated over a disease not so horrible as represented. Fish diet had no influence over leprosy. Leprosy was an endemic disease, but transmissible by heredity, but he was convinced it was not contagious. Dr. Dosabhoy Bazonjee was also of opinion that it was not contagious, and he gave instances tending to demonstrate this. Prof. Hatch mentioned a case occurring at the Jamsetjee Jeejeebhoy Hospital, of a student being accidentally inoculated, and observed that it was the *first* case in which inoculation appeared certain. There might be some doubt in the minds of many as to contagion, but even those who say leprosy is not contagious would hesitate to send their children to a school where there were lepers. He had no doubt that the bacilli of leprosy were transferable. Dr. D. B. Master observed that fish diet does not cause leprosy. Want of evidence is no proof against heredity. As to contagion, he agreed that it was not a very communicable disease. He would ask those who argue that leprosy is not contagious, if they would drink out of the Nacodo tank, or wash in the water of this tank, which is used by lepers. Scientifically, you must admit it is contagious. As regarded the leprosy scare, he agreed with former speakers. He also objected to segregation being generally applied. Dr. A. D. Modi stated that leprosy might be communicated from the ulcerated form. He considered there was great similarity between leprosy and syphilis, and would not be surprised to learn that the former is a phase of the latter. To segregate people, rich and poor, would be going too far. Dr. N. N. Katrak said the conclusion he arrived at was that leprosy was not contagious in the sense of ordinary contagious diseases. Contagion may be possible, but only to a small extent. He was not against segregation, although he believed there was some truth in what had been said regarding increase of the disease by associating all kinds of lepers together. Dr. Viccajee remarked that heredity and contagion are acknowledged factors. There ought to be a law prohibiting lepers from being *dhobees*, or dealers in provisions. Deputy Surgeon-General Hughes observed that leprosy is not as hereditary as syphilis, or even gout. We must recollect that children of lepers live in an endemic locality. The disease is contagious, but not so much so as syphilis. It is also infectious in the same way as typhoid fever—*i.e.*, it can be taken with food and water. The ulcerated form is the most dangerous, but strong and healthy people may escape. Children of lepers should be removed from the endemic area in which they are born. Pauper lepers should be segregated compulsorily. Dr. Cowasjee Hormusjee said there seemed a consensus of opinion that leprosy is in a certain form contagious, and it may be communicated to those who have no hereditary taint. Dr. Eduljee Noshorwanjee admitted that leprosy is a contagious disease, and believed those suffering from those forms of disease which are contagious should be segregated, not only for the interests of the public, but for their own, as they would be looked after and cared for. Surgeon-Major Kirtikar said leprosy was a constitutional disease of a contagious nature. If a person with only a little leprosy sore is a cook or an attendant on the sick, he may infect others without knowing it. Surgeon-Major Weir (health officer, Bombay) would not attach too much importance to one case of inoculation as sufficient to establish the disease as contagious. But it is different when there are a series of cases. There is the famous case of inoculation by Dr. Arning. There is the case referred to by Dr. Balchandra. The details of facts confirm the belief that leprosy may be conveyed to a healthy person. The speaker brought forward some statistics of leprosy in Bombay, showing that the most susceptible are low-caste Hindus. Brigade-Surgeon Gray (the Principal of the Grant Medical College) remarked that there is no satisfactory proof of leprosy increasing in Bombay, as there are no reliable statistics. People thought there were more lepers in the city than formerly, but lepers flock to where charity is most existent. Then as to lepers being a public danger, the consensus of opinion was the reverse. The public panic arose from the public not understanding the subject. Heredity appears to be proved. But as regards hereditary transmission, the question whether leprosy is more likely to be transmitted by the female than the male, requires elucidation. A point strongly urged against the contagiousness of leprosy is that hospital attendants and medical men do not contract it, but the same is the case with syphilis. Leprosy is contagious, but there are degrees of contagion, and the contagion of leprosy is not so acute as that of syphilis. Referring again to heredity, the difficulty of getting necessary information was referred to as people are reluctant to mention the fact of leprosy being in a family. The recent legislation on the subject was not intended to stamp out leprosy, but to mitigate it. It would seem that the reason why leprosy had been singled out for scare and legislation is, that the disease appeals to the sense of sight. He thoroughly agreed with Sir William Moore, who, in his recent London address, stated that the spread of education and of sanitary improvement are the true remedies of extinguishing leprosy in India. From the above somewhat meagre epitome of the proceedings of the Bombay Medical and Physical Society, the opinions of the profession in this city may be summarised as follows: That leprosy has an endemic area, that leprosy is also hereditary, that leprosy under certain conditions is contagious, that segregation of vagrant lepers is desirable, that leprosy is not caused by fish diet, that there was no reason whatever for the recent scare and agitation on the subject.

